# Exploring Age-Related Changes in Resting State Functional Connectivity of the Amygdala: From Young to Middle Adulthood

**DOI:** 10.3389/fnagi.2018.00209

**Published:** 2018-07-16

**Authors:** Ting Xiao, Sheng Zhang, Lue-En Lee, Herta H. Chao, Christopher van Dyck, Chiang-Shan R. Li

**Affiliations:** ^1^Department of Psychiatry, Yale University School of Medicine, New Haven, CT, United States; ^2^Xiangya School of Medicine, Central South University, Changsha, China; ^3^Department of Psychiatry, National Taiwan University, Taipei, Taiwan; ^4^Department of Medicine, Yale University School of Medicine, New Haven, CT, United States; ^5^VA Connecticut Healthcare System, West Haven, CT, United States; ^6^Department of Neuroscience, Yale University School of Medicine, New Haven, CT, United States; ^7^Interdepartmental Neuroscience Program, Yale University School of Medicine, New Haven, CT, United States; ^8^Beijing Huilongguan Hospital, Peking University, Beijing, China

**Keywords:** amygdala, limbic, emotion, aging, rsFC, fMRI

## Abstract

Functional connectivities of the amygdala support emotional and cognitive processing. Life-span development of resting-state functional connectivities (rsFC) of the amygdala may underlie age-related differences in emotion regulatory mechanisms. To date, age-related changes in amygdala rsFC have been reported through adolescence but not as thoroughly for adulthood. This study investigated age-related differences in amygdala rsFC in 132 young and middle-aged adults (19–55 years). Data processing followed published routines. Overall, amygdala showed positive rsFC with the temporal, sensorimotor and ventromedial prefrontal cortex (vmPFC), insula and lentiform nucleus, and negative rsFC with visual, frontoparietal, and posterior cingulate cortex and caudate head. Amygdala rsFC with the cerebellum was positively correlated with age, and rsFCs with the dorsal medial prefrontal cortex (dmPFC) and somatomotor cortex were negatively correlated with age, at voxel *p* < 0.001 in combination with cluster *p* < 0.05 FWE. These age-dependent changes in connectivity appeared to manifest to a greater extent in men than in women, although the sex difference was only evident for the cerebellum in a slope test of age regressions (*p* = 0.0053). Previous studies showed amygdala interaction with the anterior cingulate cortex (ACC) and vmPFC during emotion regulation. In region of interest analysis, amygdala rsFC with the ACC and vmPFC did not show age-related changes. These findings suggest that intrinsic connectivity of the amygdala evolved from young to middle adulthood in selective brain regions, and may inform future studies of age-related emotion regulation and maladaptive development of the amygdala circuits as an etiological marker of emotional disorders.

## Introduction

A phylogenetically old nucleus nestled deep in the temporal lobe, the amygdala plays an important role in processing negative emotions. The earliest fMRI studies reported bilateral amygdala activation to affectively negative visual stimuli (Breiter et al., 1996; Irwin et al., 1996) as well as during conditioned acquisition and extinction of fear elicited by electric shock, with a bias of right hemispheric response (LaBar et al., 1998). Other studies showed that the amygdala is involved in reward-related learning in a variety of behavioral tasks (Baxter and Murray, 2002). For instance, the amygdala showed significantly higher activation to receipt than omission of reward in a monetary reward task (Ernst et al., 2005). The amygdala may thus respond broadly to salient stimuli to support motivated behavior (Zorrilla and Koob, 2013).

In resting state fMRI the amygdala showed positive connectivity with the ventromedial prefrontal cortex (vmPFC), temporal cortex and subcortical regions, and negative connectivity with the parietal and occipital cortex (Roy et al., 2009; Veer et al., 2012). The interactions with limbic circuit support a role of the amygdala in emotion perception and memory (LeDoux, 1995). In particular, increased vmPFC along with decreased amygdala activation may reflect regulation of emotional responses to negative stimuli (Urry et al., 2006). Our earlier work demonstrated that vmPFC activity not only negatively correlated with but also Granger caused skin conductance level, an index of physiological arousal, in support of vmPFC regulation of amygdala response to saliency (Zhang et al., 2012, 2013).

Numerous studies have demonstrated age-related changes in emotion functions supported by the amygdala circuits. For instance, whereas episodic memory typically deteriorated, emotional memory appeared to be well preserved during aging (Wright et al., 2006; Ritchey et al., 2011). Compared to young people, older people remembered more positive than negative information (Charles et al., 2003), reflecting a “positivity effect,” and showed increased amygdala-mPFC/anterior cingulate cortex (ACC) connectivity during both resting state and emotion encoding (Sakaki et al., 2013). In addition, amygdala-hippocampus connectivity reversed from negative to positive during encoding of positive emotional information onward from adulthood (Addis et al., 2010). In fMRI studies of emotional memory, the effective connectivity of the amygdala was linearly correlated with memory performance in older adults (Li X. et al., 2015). Older as compared to younger adults showed stronger positive amygdala/vmPFC-hippocampus connectivities when encoding positive emotions (Addis et al., 2010). These studies suggest age-related changes in amygdala connectivity as potential neural markers of the positivity effect. On the other hand, the findings of aging on negative emotion processing appeared to be less than consistent. One study showed that amygdala functional connectivity did not differ between young and older adults in encoding negative emotional memory (Addis et al., 2010), whereas others showed increased amygdala-ventral ACC and decreased amygdala-hippocampus/posterior cortical couplings with aging during evaluation of negatively valenced pictures (Jacques et al., 2010) as well as encoding and retrieval of aversive scenes (Murty et al., 2009). There is a need to better evaluate age-related changes in amygdala connectivity, including resting-state functional connectivity (rsFC).

Age influences functional connectivity of the amygdala in relation to cognitive development. In newborns, stronger positive amygdala rsFC with the anterior insula and ventral striatum predicted higher level of fearfulness at 6 months (Graham et al., 2016). Stronger neonatal amygdala connectivity with the ventral anterior cingulate/anterior medial prefrontal cortex (mPFC) appeared to contribute to cognitive development, particularly in the context of fear related learning (Graham et al., 2016). From childhood to young adulthood in the twenties, amygdala-cortical rsFC changed dramatically (McRae et al., 2012; Gee et al., 2013), with no significant mPFC-amygdala coupling in early childhood, and positive, adult-like connectivity first emerging at around 10 years and increasing with age afterward. Similarly, small children showed no significant amygdala connectivity with the posterior cingulate/parahippocampal gyrus/cerebellum, with negative connectivity appearing only at around 10 years and becoming increasingly negative with age afterward. Amygdala connectivity with the insula/temporal/parietal regions decreased with age and turned from positive to negative at around 10 years (Gabard-Durnam et al., 2014; Alarcón et al., 2015). In contrast with these changes, amygdala rsFC with the anterior insula and ventral striatum largely remained unchanged from 4 to 23 years old (Gabard-Durnam et al., 2014). No studies to our knowledge have examined age-related changes in amygdala rsFC beyond young adulthood in neurotypical populations.

Studies have also considered sex differences in amygdala functional connectivity (Engman et al., 2016; Mareckova et al., 2016; Colich et al., 2017; Monroe et al., 2017). An earlier fMRI study reported sex by laterality interaction with higher rsFC of the right and left amygdala in men and women, respectively, with the sensorimotor cortex, striatum, and pulvinar displaying greater rsFC with the right amygdala in men and subgenual cortex, hypothalamus displaying greater rsFC with the left amygdala in women (Kilpatrick et al., 2006). As the sensorimotor cortex, striatum, and pulvinar respond to task challenges whereas the subgenual cortex and hypothalamus process self-referential information, the prevailing functional networks of the right and left amygdala appeared to be more “outwardly” and “inwardly” oriented each for men and women. These differences may have implications for sex-related differences in medical and psychiatric disorders, including irritable bowel syndrome, which is more prevalent in women and associated with greater left amygdala and subgenual cortical activation in women than in men when exposed to a visceral stressor (Naliboff et al., 2003). Together, earlier studies support the importance in examining sex differences in amygdala rsFC.

The bulk of functional connectivity studies of the amygdala employed task-based fMRI and some have examined age and sex differences. Studies of age-related changes in rsFC have largely focused on childhood and adolescence. How amygdala rsFC varies with age remains largely unknown beyond adolescence in healthy populations. Here, in a sample of 132 young and middle-aged healthy adults we assessed whole-brain rsFC of the amygdala as well as age-related changes and sex differences in amygdala rsFC. Further, considering the role of the vmPFC and ACC in emotion regulation, we employed region of interest analysis to examine whether amygdala vmPFC/ACC connectivity demonstrated age-related changes from young to middle adulthood.

## Materials and Methods

### Data Set

The data set comprised resting-state fMRI scans of 132 healthy participants (63 men, 19–55 years of age and 69 women, 20–55 years of age) obtained on a 3-Tesla Siemens Trio scanner, with one 10-min scan obtained with participants awake but with eyes closed. All participants were without major medical, neurological (including concussion that resulted in loss of consciousness) or Axis I psychiatric diagnoses and tested negative for illicit substances on the day of MR scan. Candidates who were on psychotropic medications were not invited to participate. Women who were pregnant or lactating were also excluded. All signed a written consent according to a research protocol approved by the Yale Human Investigation Committee.

### Imaging Protocol

Conventional T1-weighted spin echo sagittal anatomical images were acquired for slice localization using a 3T scanner (Siemens Trio). Anatomical images of the functional slice locations were next obtained with spin echo imaging in the axial plane parallel to the AC–PC line with TR = 300 ms, TE = 2.5 ms, bandwidth = 300 Hz/pixel, flip angle = 60°, field of view = 220 × 220 mm, matrix = 256 × 256, 32 slices with slice thickness = 4 mm and no gap. Functional, blood oxygenation level-dependent (BOLD) signals were then acquired with a single-shot gradient echo echoplanar imaging (EPI) sequence. Thirty-two axial slices parallel to the AC–PC line covering the whole brain were acquired with TR = 2000 ms, TE = 25 ms, bandwidth = 2004 Hz/pixel, flip angle = 85°, field of view = 220 × 220 mm, matrix = 64 × 64, 32 slices with slice thickness = 4 mm and no gap. There were 300 volumes for each participant. Individual subjects’ images were viewed one by one to ensure that the whole brain was covered.

### Imaging Data Processing

Brain imaging data were preprocessed using Statistical Parametric Mapping (SPM 8, Wellcome Department of Imaging Neuroscience, University College London, United Kingdom) following previous work (Kann et al., 2016; Li et al., 2017; Peterson et al., 2017; Zhang S. et al., 2017; Hu et al., 2018). Images from the first five TRs at the beginning of each trial were discarded to enable the signal to achieve steady-state equilibrium between RF pulsing and relaxation. Standard image preprocessing was performed. Images of each individual subject were first realigned (motion corrected) and corrected for slice timing. A mean functional image volume was constructed for each subject per run from the realigned image volumes. These mean images were co-registered with the high resolution structural image and then segmented for normalization with affine registration followed by non-linear transformation (Friston et al., 1995; Ashburner and Friston, 1999). The normalization parameters determined for the structure volume were then applied to the corresponding functional image volumes for each subject. Finally, the images were smoothed with a Gaussian kernel of 8 mm at Full Width at Half Maximum.

Additional preprocessing was applied to reduce spurious BOLD variances that were unlikely to reflect neuronal activity (Rombouts et al., 2003; Fox et al., 2005; Fair et al., 2007; Fox and Raichle, 2007). The sources of spurious variance were removed through linear regression by including the signal from the ventricular system, white matter, and whole brain, in addition to the six parameters obtained by rigid body head motion correction. First-order derivatives of the whole brain, ventricular and white matter signals were also included in the regression.

Cordes and colleagues suggested that BOLD fluctuations below a frequency of 0.1 Hz contribute to regionally specific BOLD correlations (Cordes et al., 2001). Thus, we applied a temporal band-pass filter (0.009 Hz < f < 0.08 Hz) to the time course in order to obtain low-frequency fluctuations, as in previous studies (Lowe et al., 1998; Fox et al., 2005; Fair et al., 2007; Fox and Raichle, 2007).

### Head Motion

As extensively investigated in Van Dijk et al. (2012), micro head motion (>0.1 mm) is an important source of spurious correlations in resting state functional connectivity analysis (Van Dijk et al., 2012). Therefore, we applied a “scrubbing” method proposed by Power and colleagues (Power et al., 2012) and successfully applied in previous studies (Smyser et al., 2010; Power et al., 2012; Tomasi and Volkow, 2014) to remove time points affected by head motions. Briefly, for every time point *t*, we computed the framewise displacement given by FD(t) = |Δd_x_(t)| + |Δd_y_(t)| + |Δd_z_(t)| + r|α(t)|+ r|β(t)| + r|γ(t)|, where (d_x_,d_y_,d_z_) and (α,β,γ) are the translational and rotational movements, respectively, and *r* ( = 50 mm) is a constant that approximates the mean distance between center of MNI space and the cortex and transform rotations into displacements (Power et al., 2012). The second head movement metric was the root mean square variance (DVARS) of the differences in % BOLD intensity I(t) between consecutive time points across brain voxels, computed as follows: DV ARS(t) = $\sqrt{\langle {\left|\text{I}(\text{t})-\text{I}\left(\text{t}-1\right)\right|}^{2}\rangle }$ where the brackets indicate the mean across brain voxels. Finally, to compute each subject’s correlation map, we removed every time point that exceeded the head motion limit FD(*t*) > 0.5 mm or DVARS(*t*) > 0.5% (Power et al., 2012; Tomasi and Volkow, 2014). On average, 1% of the time points were removed across subjects.

### Seed Based Correlation and Group Analyses

The amygdala masks were obtained from the Automated Anatomic Labeling (AAL) atlas with 116 anatomical masks in MNI space (Tzourio-Mazoyer et al., 2002) and comprised 3,744 mm^3^ or approximately 138 voxels (Brabec et al., 2010). The BOLD time courses were averaged spatially over all voxels each for the left (L) and right (R) amygdala, and for L and R amygdala combined (L+R). For individual subjects, we computed the correlation coefficient between the averaged time course of each seed region and the time courses of all other brain voxels. To assess and compare the rsFC, we converted these image maps, which were not normally distributed, to z score maps by Fisher’s z transform (Jenkins and Watts, 1968; Berry and Mielke, 2000): z = 0.5log_e_[(1 + r)/(1 − r)]. The Z maps were used in group random effect analyses.

**FIGURE 1:**
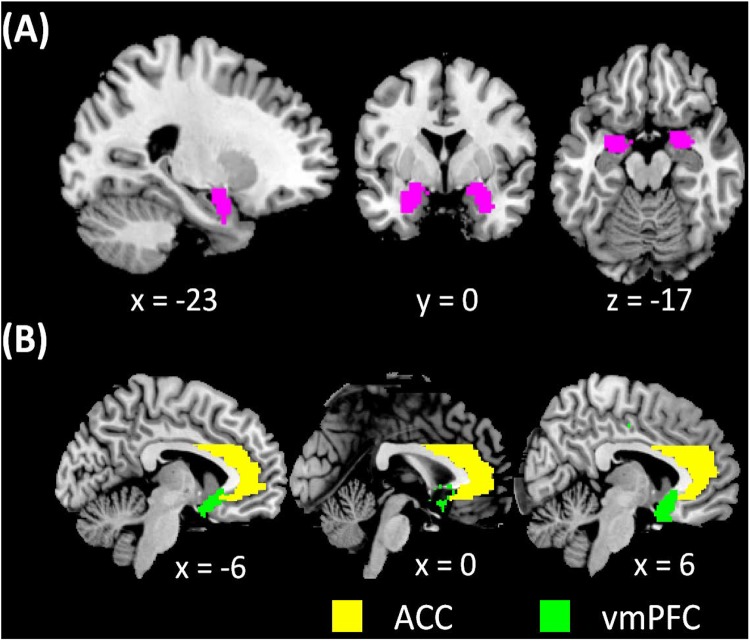
**(A)** amygdala masks used in whole-brain analysis of resting state functional connectivity (rsFC) and **(B)** vmPFC and ACC masks used in region of interest analysis of amygdala rsFC.

For group analyses, we first examined sex differences in whole-brain connectivity with a two-sample *t*-test and in age-related changes in connectivities by comparing the age regressions between men and women. At *p*<0.001, uncorrected, there were no significant clusters showing sex differences in either analysis, with L, R, or L+R as the seed region (**Figure 1A**). Thus, we performed a one-sample t test to show whole-brain rsFC for L+R seeds, and regression analysis with age on the Z maps of L, R as well as L+R seeds, for men and women combined as well as for men and women separately. In addition to whole-brain analysis, we also focused on the vmPFC and ACC with small volume correction to examine the results. The vmPFC and ACC masks comprised the rostral, perigenual, and subgenual ACC as well as the most posterior ventral part of the medial orbitofrontal cortex (**Figure 1B**). All results were evaluated at voxel *p*<0.001, uncorrected in combination with a cluster *p*<0.05, corrected for familywise error of multiple comparisons, following current reporting standards. For the clusters showing an age effect in men or women alone, we examined sex (men vs. women) differences in the slope of regression of connectivity effect size (*z* value) vs. age (Zar, 1999).

## Results

### Whole-Brain Amygdala Connectivity

**FIGURE 2:**
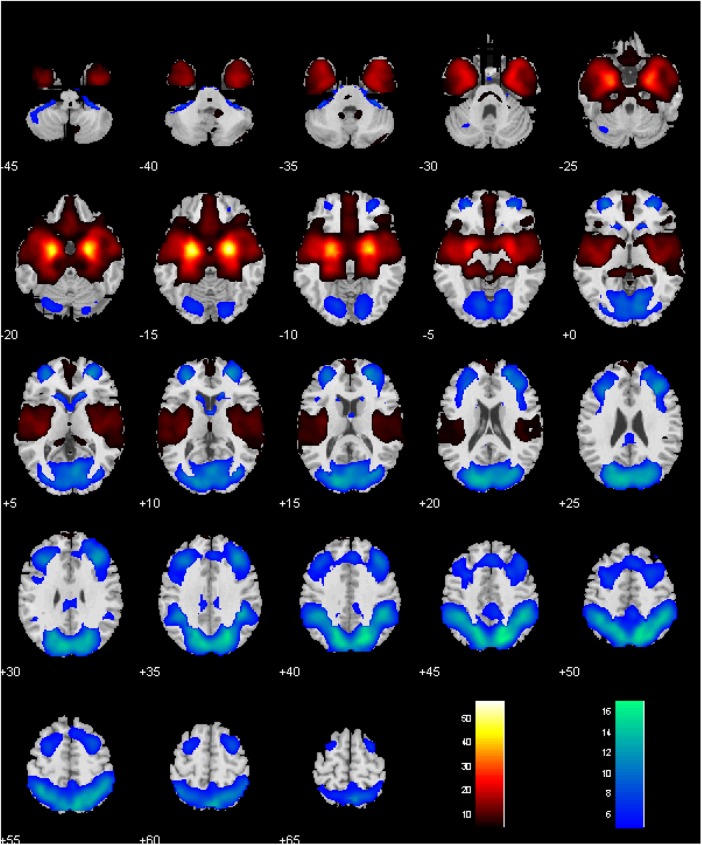
Whole-brain rsFC of the amygdala, with left- and right- hemispheric seeds combined. Voxel *P* < 0.05 corrected for family-wise error of multiple comparisons. Clusters with positive and negative connectivity are shown in warm and cool color, respectively. Color bar shows *T*-value of voxels. Neurological orientation: right = right.

Men and women did not show differences in amygdala connectivity of left-hemispheric (L), right-hemispheric (R), or both seeds combined (L+R), at voxel *p*< 0.001, uncorrected. Thus, a one-sample *t*-test of men and women combined was used to evaluate whole brain connectivity of the amygdala. With L+R seed, the amygdala showed positive connectivity with subcortical areas including the lentiform nucleus and part of the thalamus, insula, inferior temporal cortex, hippocampus, vmPFC, medial orbitofrontal cortex (OFC), gyrus rectus, and frontopolar cortex, and negative connectivity with the lateral/medial frontal and posterior parietal areas, precuneus, part of the posterior cingulate gyrus, as well as the occipital cortex, lateral OFC, and caudate head (**Figure 2**) (Roy et al., 2009; Veer et al., 2012).

### Age-Related Changes in Amygdala Connectivity

**FIGURE 3:**
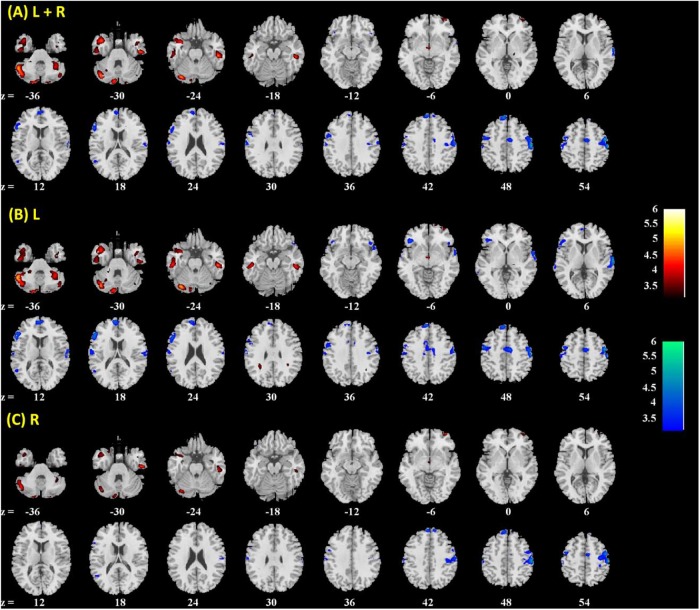
Brain regions showing positive (warm color) and negative (cool color) age correlation with **(A)** left + right, **(B)** left, and **(C)** right amygdala connectivity in men and women combined. Threshold; *p* < 0.001 uncorrected. Clusters with *p* < 0.05 FWE-corrected are summarized in **Table 1**. Color bar shows *T*-value of voxels. Neurological orientation: right = right.

**FIGURE 4:**
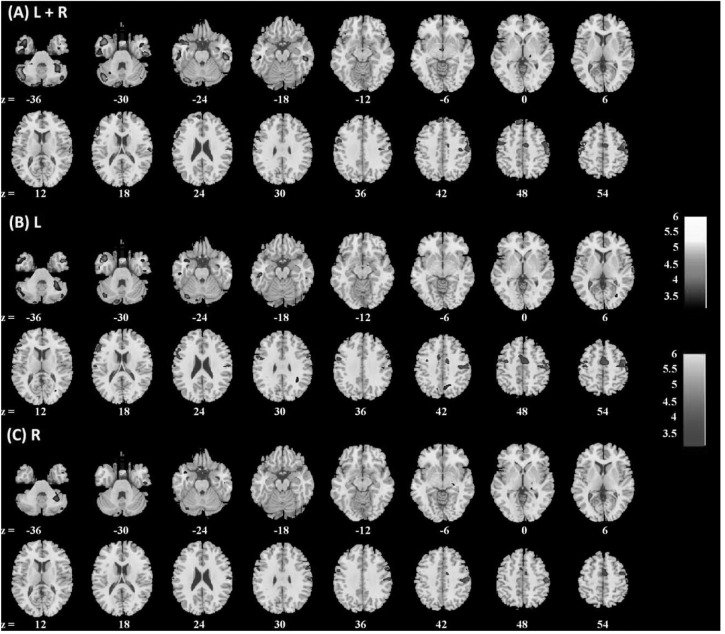
Brain regions showing positive (warm color) and negative (cool color) age correlation with **(A)** left + right, **(B)** left, and **(C)** right amygdala connectivity in men only. Threshold; *p*<0.001 uncorrected. Clusters with *p* < 0.05 FWE-corrected are summarized in **Table 2**. Color bar shows *T*-value of voxels. Neurological orientation: right = right.

**FIGURE 5:**
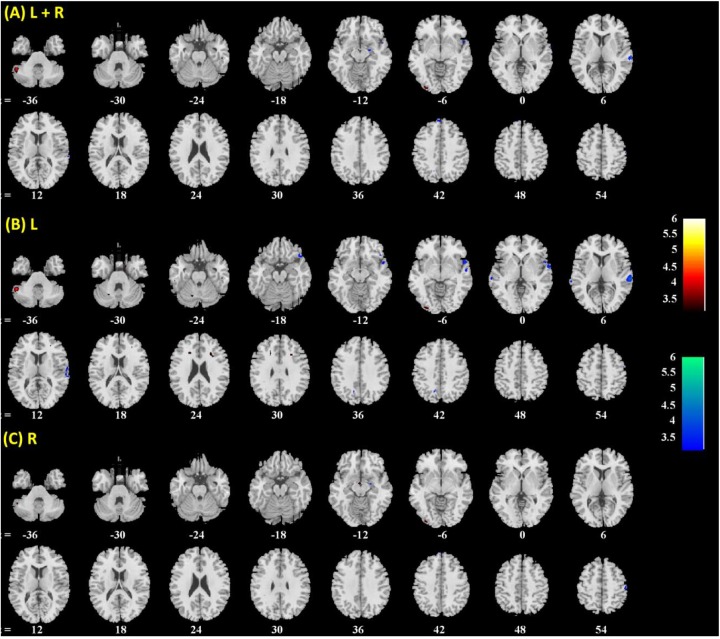
Brain regions showing positive (warm color) and negative (cool color) age correlation with **(A)** left + right, **(B)** left, and **(C)** right amygdala connectivity in women only. Threshold; *p* < 0.001 uncorrected. Clusters with *p* < 0.05 FWE-corrected are summarized in **Table 3**. Color bar shows *T*-value of voxels. Neurological orientation: right = right.

Amygdala rsFC with the bilateral cerebellum and left temporal gyrus increased with age and amygdala rsFC with the right somatomotor cortex and left superior/inferior frontal gyrus decreased with age in men and women combined (**Figure 3** and **Table 1**). We also examined age-related changes in amygdala rsFC in men and women separately. Overall, men as compared to women appeared to demonstrate more age-related changes in rsFC and the patterns of changes were similar to those observed for men and women combined (**Figures 4**, **5** and **Tables 2**, **3**). In slope tests, men and women showed a trend-level difference in amygdala (L+R) rsFC with the right cerebellum (*t* = 2.84, *p* = 0.0053), with correction for multiple tests (*p* = 0.05/12 = 0.0042). In **Figures 3**–**5** and **Tables 1**–**3** we showed the results for L+R, L, and R amygdala seeds for women and men combined and separately.

**Table 1:** Regions showing age effect on the functional connectivity for amygdala (R+L, R, and L) in men and women combined.

Volume	Peak voxel	MNI coordinate			Side	Identified brain region
(mm^3^)	(Z)	x	y	z		
**Amygdala (positive correlation with Age)**						
10,854	5.37	−51	−52	−41	L	Cerebellum
8,937	4.64	−48	−16	−35	L	Superior/Middle/Inferior Temporal G
3,294	4.62	51	−25	−23	R	Cerebellum
8,478	4.43	42	−61	−50	R	Cerebellum
**Amygdala (negative correlation with Age)**						
34,452	5.60	57	−22	52	R	Precentral G/Inferior Precentral S
	5.48	42	−10	64	R	Superior Precentral/Postcentral G
	5.33	−12	−1	70	L	Superior Frontal G
3,456	4.03	−57	23	13	L	Inferior Frontal G
**Left Amygdala (positive correlation with Age)**						
15,984	5.84	−51	−52	−41	L	Cerebellum
9,909	4.58	−51	−25	−20	L	Cerebellum
3,213	4.57	51	−28	−20	R	Cerebellum
9,261	4.24	36	−49	−38	R	Cerebellum
**Left Amygdala (negative correlation with Age)**						
43,146	5.56	−12	−1	73	L	Superior Frontal G
	5.12	45	−10	61	R	Superior Precentral/Postcentral G
	4.96	51	−7	55	R	Inferior Precentral S
7,641	4.89	69	−13	7	R	Superior Temporal G
5,724	4.39	−6	47	46	L/R	Anterior Cingulate G
**Right Amygdala (positive correlation with Age)**						
6,750	4.32	−51	−55	−38	L	Cerebellum
**Right Amygdala (negative correlation with Age)**						
19,251	5.68	39	−10	64	R	Superior Precentral/Postcentral G
	5.39	57	−22	52	R	Inferior Precentral/Postcentral G
3,672	3.99	−42	−1	56	L	Superior Precentral S

*Voxel p < 0.001 uncorrected and cluster-level p < 0.05, FWE; R, right; L, left; G, gyrus; S, sulcus.*

**Table 2:** Regions showing age effect of the functional connectivity for amygdala (R+L, R, and L) in Men.

Volume	Peak voxel	MNI coordinate			Side	Identified brain region
(mm^3^)	(Z)	x	y	z		
**Amygdala (positive correlation with Age)**						
5,022	4.36	−51	−55	−41	L	Cerebellum
5,697	4.29	33	−49	−38	R	Cerebellum
**Amygdala (negative correlation with Age)**						
27,189	4.80	57	−22	52	R	Precentral G
	4.82	12	4	70	L	Superior Frontal G/Anterior Cingulate G/SMA
3,564	3.90	54	2	37	L	Inferior Precentral G
**Left Amygdala (positive correlation with Age)**						
5,238	4.72	−51	−55	−44	L	Cerebellum
5,130	4.28	33	−49	−38	R	Cerebellum
3,051	4.12	−39	11	−32	L	Superior Temporal G
**Left Amygdala (negative correlation with Age)**						
27,270	4.90	−6	5	67	L	Superior Frontal G/Anterior Cingulate G/SMA
	4.65	−12	−1	70	L	Superior Precentral S
	4.63	57	−22	52	R	Precentral G
6,642	4.32	−57	14	4	L	Inferior Frontal G
**Right Amygdala (positive correlation with Age)**						
none						
**Right Amygdala (negative correlation with Age)**						
10,962	4.46	27	−7	67	R	Superior Frontal G/Superior Precentral S
	4.05	9	−4	61	R	SMA
4,185	4.31	57	−22	52	R	Precentral G

*Voxel p < 0.001 uncorrected and cluster-level p < 0.05, FWE; R, right; L, left; G, gyrus; S, sulcus; SMA, supplementary motor area.*

**Table 3:** Regions showing age effect of the functional connectivity for amygdala (R+L, R, and L) in Women.

Volume	Peak voxel	MNI coordinate			Side	Identified brain region
(mm^3^)	(Z)	x	y	z		
**Amygdala (positive correlation with Age)**						
None						
**Amygdala (negative correlation with Age)**						
None						
**Left Amygdala (positive correlation with Age)**						
None						
**Left Amygdala (negative correlation with Age)**						
3,699	4.41	66	−25	10	R	Superior Temporal G
	3.97	54	11	−5	R	Superior Temporal G
**Right Amygdala (positive correlation with Age)**						
None						
**Right Amygdala (negative correlation with Age)**						
None						

*Voxel p < 0.001 uncorrected and cluster-level p < 0.05, FWE; R, right; L, left; G, gyrus.*

In region of interest analysis, no clusters within the vmPFC/ACC mask showed significant age-related changes in amygdala rsFC at voxel *p*<0.001, uncorrected.

## Discussion

### Whole Brain Functional Connectivity of the Amygdala

Consistent with previous studies (Roy et al., 2009; Veer et al., 2012), the present work showed that the amygdala is positively connected with the vmPFC, inferior frontal cortex, inferior temporal cortex, hippocampus, parahippocampal gyrus, insula, rolandic fissure, postcentral gyrus, subcortical areas, including the lentiform nucleus and part of the thalamus, and negatively connected with the lateral/medial frontal areas, parietal cortex, occipital cortex, posterior cingulate cortex, precuneus, and cerebellum. We highlighted age-related changes in amygdala rsFC in the discussion below.

### Amygdala rsFC With the Cerebellum

A recent study of independent component analysis of resting state data identified a network that included deep cerebellar nuclei, the cerebellar cortex, basolateral amygdala, insula, and claustrum (Habas, 2018). The amygdala showed a negative rsFC with the cerebellum in one sample *t*-test, and amygdala-cerebellum rsFC was positively correlated with age, becoming less negative from young to middle adulthood. A study of participants 4–23 years old showed amygdala rsFC with the cerebellum in negative correlation with age, with the rsFC becoming more negative after around 10 years (Gabard-Durnam et al., 2014). Together, these findings suggest that amygdala connectivity with the cerebellum demonstrated age-related curvilinear changes from early childhood to mid-adulthood.

Anatomically connected with the amygdala, the cerebellum is involved in emotional experience and regulation (De Bellis et al., 2002; Turner et al., 2007) and other cognitive processes (Schmahmann, 1991; Timmann et al., 2010). For instance, the cerebellum responded to moral judgment (Harada et al., 2009; Leutgeb et al., 2016), empathy (Lang et al., 2011), and fear (Sacchetti et al., 2005; Hakamata et al., 2017). In adult rats, pharmacologically blocking activity of the amygdala or cerebellar vermis led to long-term fear memory loss (Sacchetti et al., 2007). Pharmacological inactivation of the central amygdala reduced stimulus-evoked and learning-related neuronal activity in the cerebellar interpositus nuclei during acquisition and retention of eyeblink conditioning in rats (Farley et al., 2016). Classical conditioning is thought to proceed through two successive stages: fast emotional conditioning followed by slower motor conditioning, each supported by the amygdala and cerebellum, respectively. That is, conditioned motor response depends on the integrity of the cerebellar interpositus nucleus in promoting the extinction of amygdala response (Mintz and Wang-Ninio, 2001). Further, altered amygdala-cerebellum functional connectivity has been implicated in emotional disorders (Liu et al., 2015). More research is needed to investigate how age-related changes in amygdala cerebellar connectivity relate to affective processing and emotional well-being over the life span.

### Amygdala rsFC With the Temporal Cortex

The amygdala, insula and temporal cortex are critical to empathetic experience (Shdo et al., 2017), and the temporal pole plays an instrumental role in supporting long-term social and emotional memory (Olson et al., 2007). Age-related increase in amygdala rsFC with the temporal cortex may be associated with older adults’ reliance on general information rather than perceptual details in emotion recognition (Kalpouzos et al., 2012). Disruption of this circuit was related to late life depression (Li W. et al., 2015), and neurofeedback training altered amygdala temporal pole connectivity during positive autobiographical recall in depression patients (Young et al., 2018). Along with these earlier studies, the current results suggest the importance of amygdala temporal pole circuit in emotional memory and regulation during aging.

### Amygdala rsFC With the Dorsal Medial Prefrontal Cortex (dmPFC)

The dmPFC, including the ACC and supplementary motor area (SMA), showed negative rsFC with the amygdala, and amygdala-dmPFC connectivity become more negative from young to middle adulthood, essentially reversing the trend from childhood to young adulthood (Alarcón et al., 2015).

The dmPFC is involved in regulating emotion, and planning and monitoring behavioral responses to affective stimuli (Morecraft et al., 2004; Nachev et al., 2008; Vrtička et al., 2011; Dörfel et al., 2014; Picó-Pérez et al., 2017). As with viewing happy faces, electrical stimulation of the SMA elicited merriment (Krolak-Salmon et al., 2006). When exposed to visual images of spiders, amygdala-SMA connectivity increased in association with fear (Goossens et al., 2007). In an action selection task, the amygdala responded to emotional salience, and lower SMA activity was associated with motor action (Voon et al., 2010). In a modified stop-signal task (Sagaspe et al., 2011), emotional cues (fearful faces) during stop trials engaged the amygdala and SMA, suggesting that amygdala SMA interaction in the context of fearful emotion may lead to changes in the suppression of ongoing actions.

The functional relationship between amygdala and dmPFC can also be considered in the context of two emotion regulatory mechanisms – cognitive reappraisal and expressive suppression. Cognitive reappraisal supports better control prior to the generation of emotion. In contrast, expressive suppression reflects poor control of emotion and ongoing expression after the onset of emotional behavior. A recent study reported amygdala-SMA rsFC in negative and positive correlation each with reappraisal and suppression score, suggesting its distinct roles in emotional regulation strategies (Picó-Pérez et al., 2017). As one ages, more negative amygdala-dmPFC connectivity may reflect top-down prefrontal regulation of amygdala response, a process critical to emotion appraisal and regulation.

Notably, compared with healthy individuals, the functional connectivity between the amygdala and SMA increased in Parkinson’s disease (Yu et al., 2013), conversion disorder (Voon et al., 2010), fibromyalgia (Kim et al., 2013), and non-suicidal self-injurious behavior (Schreiner et al., 2017), all of which implicate emotion regulation dysfunction. Age-related enhancement of negative amygdala-dmPFC connectivity may support emotion regulation and its dysfunction would likely lead to emotional disorders.

### Amygdala rsFC With the Somatomotor Cortex

The amygdala is anatomically connected with motor and premotor structures (Macchi et al., 1978; Amaral and Price, 1984; Jürgens, 1984; Sripanidkulchai et al., 1984). Diffusion tensor imaging identified connections between the amygdala and sensory-motor areas (Grezes et al., 2014). A recent rsFC study employed independent component analysis and characterized a network that comprised the amygdala and somatomotor and premotor cortex (Toschi et al., 2017). Fear conditioning elevated auditory startle response and motor evoked potentials (Gökdemir et al., 2018). With transcranial magnetic stimulation, Borgomaneri and colleagues demonstrated that behavioral inhibition sensitivity was associated with enhanced motor cortical suppression in response to fearful body expressions (Borgomaneri et al., 2017). Together, these studies supported a functional link of the motor cortex to emotional behavior.

We showed that age was associated with decreased amygdala somatomotor cortical rsFC, in accord with studies reporting age-related changes in emotional motor activities. For instance, pleasant compared to unpleasant visual stimulation facilitated motor response in young but not older adults (Vernazza-Martin et al., 2017; see Hälbig et al., 2011 for contrasting findings). Aging was associated with decreased response to emotionally arousing stimuli in the occipital and temporal visual cortices, inferior parietal cortex, and supplementary motor area (Kehoe et al., 2013). These findings may be considered along older adults’ ability to deploy cognitive resources, rather than rushing into a response, when facing emotional situations (Mather, 2016).

### Amygdala rsFC With the Anterior Cingulate and Ventromedial Prefrontal Cortex

Many studies have implicated functional interaction between the amygdala and the ACC and vmPFC during emotion regulation. VMPFC regulated amygdala responses during fear and anxiety (Gee et al., 2013; Vink et al., 2014; Wu et al., 2016), whereas GABAergic projection from the amygdala to vmPFC supported reward processing (Seo et al., 2016). Amygdala vmPFC rsFC predicted fear extinction following cue exposures (Feng et al., 2016). In particular, older people demonstrated differences in this regulatory mechanism. Older but not young people who tended to remember more positive than negative information showed increased amygdala-mPFC/ACC connectivity during both resting state and emotion encoding (Sakaki et al., 2013). In contrast to previous reports of increased amygdala rsFC with the vmPFC from childhood to young adulthood (Gabard-Durnam et al., 2014; Alarcón et al., 2015), the current findings showed that amygdala rsFC with the ACC and vmPFC did not vary with age from young and middle adulthood. It is possible that age impacts this functional circuit only when individuals were engaged in a cognitive task. Alternatively, the amygdala vmPFC/ACC circuit may have “matured” through young adulthood. More research is needed to resolve this issue as well as to investigate how amygdala mPFC rsFC may change beyond middle adulthood and how these age-related changes influence emotional experience. In particular, amygdala connectivity with the vmPFC in response to emotional challenges shifted from positive in early childhood to negative in adolescence at around 15 years, and became increasingly negative through age 25 years (Gee et al., 2013; Vink et al., 2014; Wu et al., 2016). In accord with a regulatory role of the vmPFC, amygdala response to emotional faces decreased from 8 to 24 years (Swartz et al., 2014; Vink et al., 2014). Heart rate variability was associated with stronger mPFC-amygdala rsFC (Sakaki et al., 2016). These findings suggest that the vmPFC may exert top-down control over amygdala in emotional processing, which grows in strength from adolescence to young adulthood, in accord with increasing structural connectivity through the uncinate fasciculus, a major white-matter tract connecting the amygdala and vmPFC (Ford and Kensinger, 2014; Swartz et al., 2014). More studies are needed to address how resting state and task-related amygdala vmPFC connectivities evolve throughout the life span and whether these connectivity changes account for differences in emotion regulation, such as the “positivity effect,” as observed in older adults.

## Conclusions and Limitations

These findings suggest that intrinsic connectivity of the amygdala evolved from young to middle adulthood in selective brain regions, with amygdala rsFCs with the cerebellum and temporal gyri positively correlated with age, and rsFCs with the dorsal medial prefrontal cortex and somatomotor cortex negatively correlated with age. Amygdala rsFC with the anterior cingulate and vmPFC did not show age-related changes in adults from 19 to 55 years. The current findings extend the literature which has thus far focused on adolescence and young adulthood and may facilite future research of amygdala functional dysconnectivity as etiological markers of psychiatric and neurological conditions (Chen et al., 2017; Hafeman et al., 2017; Zhang J. et al., 2017; Hu et al., 2018; Tang et al., 2018; Wheeler et al., 2018).

A few limitations should be considered. First, the study did not include older individuals and thus the findings should be considered as specific to young through middle adulthood. Second, we used a kernel of 8 mm in smoothing. Although our earlier work, including those of subcortical structures smaller than the amygdala, suggested that kernel size did not influence seed-based connectities characterized by simple correlations (Kline et al., 2016; Zhang et al., 2016), other studies have demonstrated the advantage of higher resolution in revealing connectivity features, such as small-world network properties (Hayasaka and Laurienti, 2010). Third, the participants were not assessed for personality traits or neuropsychological performance. Studies combining imaging and these assessments would provide an opportunity to evaluate the relationship of depression and anxiety trait as well as emotion regulation and amygdala connectivity. Finally, correlation analysis does not reflect directional interactions between brain regions. Research combining behavioral tasks with tools such as dynamic causal modeling and Granger causality analysis may reveal directional functional connectivities of the amygdala (Ide and Li, 2011; Hu et al., 2015; Zhang et al., 2015; Sato et al., 2017; Zhang Y. et al., 2017; Velichkovsky et al., 2018; Wang et al., 2018). This research may also benefit from studies combining functional and spectroscopy imaging of neurotransmitters (Delli Pizzi et al., 2017a,b) and animal work that employs pharmacological manipulations to investigate the neurochemical mechanisms underlying amygdala interaction with other brain regions (Andolina et al., 2013; Chang and Ho, 2017; Chang and Grace, 2018).

## Ethics Statement

This study was carried out in accordance with a protocol approved by the Yale University Human Investigation Committee. All subjects gave written informed consent in accordance with the Declaration of Helsinki.

## Author Contributions

TX, CvD, and C-SL contributed to the design of the study. SZ performed data analyses. All authors contributed to literature review, writing, and critical revision of the manuscript.

## Conflict of Interest Statement

The authors declare that the research was conducted in the absence of any commercial or financial relationships that could be construed as a potential conflict of interest.
